# Supplementary material for: Lessons from Fraxinus, a crowd-sourced citizen science game in genomics
Source: eLife. 2015 Jul 29;4:e07460. doi: 10.7554/eLife.07460 (PMC4517073; doi:10.7554/eLife.07460)
Supplement: Supplementary file 1. — Table S1: details of alignment answers received for the Fraxinus version 1. Table S2: comparison of the alignments that were aligned differently to software by players. Table S3: details of news articles, press releases, and social network mentions promoting Fraxinus game. Table S4: details about most active players contributing to Fraxinus. Table S5: list of players agreed to be included as authors under ‘Fraxinus Players’. DOI: http://dx.doi.org/10.7554/eLife.07460.016 [file elife07460s001.pdf]

Supplemental File 1.

Rallapalli et al., Fraxinus: a citizen science game where players improve software DNA alignments & a model for strategising in crowdsourcing

Table S1. Details of alignment answers received for the Fraxinus version 1

Category	all	SNP	INDEL
Total no. of puzzles	10087	9022	1065
Total no. of alignments	154038	138759	15279
Total no. of empty alignments	35921	33214	2707
Selected puzzles for analysis	7620	6798	822
No. of alignments for selected puzzles	117471	105647	11824
Puzzles - all high score players aligned differently to software	4701	3997	704
Puzzles - few high score players aligned differently to software	154	99	55
Puzzles - no high score players aligned differently to software	2765	2702	63

Table S2. Comparison of the alignments that were aligned differently to software by players

Type	Aligner	Puzzles		
		all	SNP	INDEL
Identity	Player	15.26	12.32	28.75
	Both Equal	78.37	80.77	67.36
	Software	6.37	6.91	3.89
Score	Player	18.09	14.97	32.40
	Both Equal	78.19	80.95	65.51
	Software	3.73	4.08	2.09

Table S3. Details of news articles, press releases and social network mentions promoting Fraxinus game

Type	Description	url	Date
News articles	BBC & UK newspapers	http://www.bbc.co.uk/news/science-environment-23635094	13/08/2013
	The Scientist	http://www.the-scientist.com/?articles.view/articleNo/37121/title/Gamers-to-Fight-Tree-Ailment/	21/08/2013
	BBC	http://www.bbc.co.uk/news/science-environment-24134879	23/09/2013
	TIGA Awards day	http://awards13.tiga.org/awards_ceremony.php	06/11/2013
	BBC countryfile	http://www.bbc.co.uk/programmes/b03j4y40	10/11/2013
	The Guardian	http://www.theguardian.com/technology/2014/jan/25/online-gamers-solving-sciences-biggest-problems	25/01/2014
Social network & Institute articles	TSL/JIC	http://news.jic.ac.uk/2013/08/gamers-to-join-ash-dieback-fight-back/	12/08/2013
	TSL/JIC	http://news.jic.ac.uk/2013/08/fraxinus-fever/	30/08/2013
	Twitter (games for change competition)	https://twitter.com/TheSainsburyLab/status/391161357036711937	18/10/2013
	Facebook (BBC countryfile)	https://www.facebook.com/photo.php?fbid=1448568202036769	08/11/2013
	Facebook (Game Update)	https://www.facebook.com/fraxinusgame/posts/1488147001412222	06/02/2014

Table S4. Details about most active players contributing to Fraxinus

UserID	Days Since Joining	Play Days	Inactive Days	Activity Ratio
12269	350	332	18	18.44
7650	355	318	37	8.59
20536	330	279	51	5.47
8970	354	282	72	3.92
1315	343	271	72	3.76
8907	115	80	35	2.29
3792	356	247	109	2.27
14244	349	222	127	1.75
737	355	206	149	1.38
638	356	196	160	1.23
5315	348	164	184	0.89
12785	350	163	187	0.87
9176	355	158	197	0.80
12295	350	139	211	0.66
859	325	117	208	0.56
25113	145	52	93	0.56
6166	357	127	230	0.55
761	353	123	230	0.54
16183	347	120	227	0.53

Table S5. List of players agreed to be included as authors under ”Fraxinus Players”

Player Name[†]	Score
Colin Higgs	11146
Gonçalo José Martins Cabrita	5502
Lucy Lewis	3962
Jayne Shardlow	2867
Sue Bell	1840
Lenka Habetinova	304
Natalia Martinkova	77
Terri Grassby	35
Kate Ure	24
Robert Waterman	7
Amanda Riley	1
Miranda Stoneham	1
Eileen Thomas	1

[†] Fraxinus privacy policy is available at `https://oadbproject.tsl.ac.uk/terms.html`.
